# *Lactobacillus plantarum* PL-02 Supplementation Combined With Resistance Training Improved Muscle Mass, Force, and Exercise Performance in Mice

**DOI:** 10.3389/fnut.2022.896503

**Published:** 2022-04-27

**Authors:** Wen-Ling Yeh, Yi-Ju Hsu, Chin-Shen Ho, Hsieh-Hsun Ho, Yi-Wei Kuo, Shin-Yu Tsai, Chi-Chang Huang, Mon-Chien Lee

**Affiliations:** ^1^Department of Orthopedic Surgery, Lotung Poh-Ai Hospital, Luodong, Taiwan; ^2^Department of Orthopedic Surgery, Chang Gung Memorial Hospital, Taoyuan, Taiwan; ^3^Graduate Institute of Sports Science, National Taiwan Sport University, Taoyuan, Taiwan; ^4^Department of Research and Design, Bioflag Biotech Co., Ltd., Tainan, Taiwan

**Keywords:** *Lactobacillus plantarum* PL-02, probiotic, muscle mass, exercise, training

## Abstract

Increasing numbers of researchers are investigating the benefits of probiotics in enhancing exercise performance and verifying the role of the gut–muscle axis. In our previous study, *Lactobacillus plantarum* PL-02 improved exercise performance and muscle mass. Therefore, the purpose of this study was to investigate whether supplementation with PL-02 combined with resistance training has a synergistic effect on exercise performance and muscle mass. All the animals were assigned into four groups (*n* = 8/group): a sedentary control with normal distilled water group (vehicle, *n* = 8); PL-02 supplementation group (PL-02, 2.05 × 10^9^ CFU, *n* = 8); resistance training group (RT, *n* = 8); PL-02 supplementation combined with resistance training group (PL-02 + RT, 2.05 × 10^9^ CFU, *n* = 8). Supplementation with PL-02 for four consecutive weeks combined with resistance exercise training significantly improved the grip strength and the maximum number of crawls; increased the time of exhaustive exercise; significantly reduced the time required for a single climb; and reduced the lactate, blood ammonia, creatine kinase, and blood urea nitrogen produced after exercise (*p* < 0.05). In addition, it produced substantial benefits for increasing muscle mass without causing any physical damage. In summary, our findings confirmed that PL-02 or RT supplementation alone is effective in improving muscle mass and exercise performance and in reducing exercise fatigue, but the combination of the two can achieve increased benefits.

## Introduction

Skeletal muscle is one of the most important tissues in the human body, accounting for approximately 40% of body weight, so maintaining skeletal muscle mass and strength is critical. Resistance training is an effective anabolic stimulus that increases muscle mass and strength in men and women of all ages (1). Regular resistance training (RT) can also improve exercise performance and health (2). Appropriate RT prescriptions and strategies can effectively prevent and improve cardiovascular disease (3), diabetes (4), and arthritis (5), and can improve and delay the risk of sarcopenia and frailty-related diseases in the elderly (6). For athletes, improving muscle strength and mass through RT can help to develop increased explosive power and power output, and can improve muscle endurance to maintain exercise performance during competition (7). Therefore, formulating different training modes, times, intensities, and frequencies for different needs is an important method of optimizing resistance training (8). In recent years, sports nutrition supplements, when combined with exercise training intervention, more effectively repairs muscles, increases muscle protein synthesis (MPS) rate, reduces exercise injuries, and improves exercise performance (9, 10). In addition to the common high-protein creatine diet, a growing body of evidence supports the idea of a gut–muscle axis, implying a link between muscle mass and strength performance and gut microbes (11). Probiotics are a safe dietary supplement that improves the richness and abundance of gut microorganisms (12).

Although the mechanisms underlying the relationship between gut microbes and muscle have not been fully defined, researchers have found that exercise training can alter the composition of the gut microbiota, possibly due to energy demand and use (13). Gut microbes may also stimulate insulin-like growth factor-1 (IGF-1) to increase skeletal muscle mitochondrial levels, promote skeletal muscle cell synthesis (14), or maintain or improve muscle mass by increasing glycogen storage using short-chain fatty acids (SCFA) (15). Many factors can cause changes in the gut microbiome, such as diet and exercise. Diet and exercise can positively change the gut microbiota (16). In addition to exercise training, supplementation with probiotic is another method to affect the gut microbiota. Probiotics are defined as live microorganisms that, when administered in sufficient doses, confer a health benefit to the host (17). Currently, lactic acid bacteria (LAB) and bifidobacteria are the main probiotic species. The health benefits of probiotics include the modulation of immune responses, maintenance of the gut barrier, and the antagonism of pathogen adhesion to host tissues (18–20). However, different strains have different levels of efficacy, and probiotics vary in their ability to colonize the gastrointestinal (GI) tract, clinical efficacy, and the type and extent of health benefits they provide to different hosts (21). In recent years, increasing numbers of studies have shown that probiotic supplementation can effectively improve exercise endurance performance, increase glycogen storage, and delay fatigue. Among them, *Lactobacillus plantarum*, *Bifidobacterium longum* subsp. *longum*, and *Lactobacillus salivarius* subsp. *salicinius* significantly improve muscle mass and muscle strength. In our previous research, *L. plantarum* TWK10, *Bifidobacterium longum* subsp. *longum* OLP-01, and *Lactobacillus salivarius* subsp. *salicinius* SA-03 supplementation was found to be effective not only in increasing muscle mass and grip strength in animals (22, 23), but also in improving body composition and exercise performance in healthy adults (24–26).

In the current study, we used *L. plantarum* PL-02 in combination with resistance training to evaluate the effect on improving exercise performance and increasing muscle mass in mice. This strain is a human-derived probiotic, which was screened from the gut microbiota of a women’s weightlifting Olympic gold medalist. In our previous study, we confirmed that supplementation with PL-02 without exercise training effectively improved muscle strength, exercise endurance performance, glycogen storage, and muscle mass in mice, and delayed and reduced the fatigue index after exercise (27). Therefore, we aimed to explore the effects of supplementary PL-02 and resistance exercise training on improving muscle mass, strength, exercise performance, and physical fitness.

## Materials and Methods

### Materials, Animals, and Study Design

We screened *L. plantarum* (PL-02) from the feces of the Olympic women’s 48 kg weightlifting gold medalist Wei-ling Chen and collected fecal specimens using a fecal preservation kit. We analyzed the specimens after 1–4 days at 37^°^C using glucose-molasses medium (GMM) under three different oxygen conditions (aerobic, facultative anaerobic, and obligate anaerobic). The cultured intestinal flora was extracted using 16S rDNA sequencing, and the Food Industry Research and Development Institute (Hsinchu, Taiwan) confirmed that the isolate was *L. plantarum*. The probiotics supplementation (PL-02) was provided by Bioflag Biotech Co., Ltd. (Tainan, Taiwan), and the PL-02 was preserved in Food Industry Research and Development Institute (Hsinchu, Taiwan) and China General Microbiological Culture Collection Center (Beijing, China), the deposition number of PL-02 were BCRC-911012 and CGMCC-20485, respectively. The viable cell number of PL-02 cells was 1.07 × 10^11^ CFU/g. We dissolved the strain powder in phosphate-buffered saline (PBS, pH. 7.2) prior to supplementation. The Institutional Animal Care and Use Committee (IACUC) of the National Taiwan Sport University approved the animal experimentation and procedure (IACUC no. 11008). All mice were housed in the Animal Facility of the Institute of Sports Science, National Taiwan Sports University, and maintained under stable photoperiod, temperature, and humidity conditions (12 h light/12 h dark cycle, 22 ± 2°C, and 60–70%, respectively). A standard laboratory diet (No. 5,001; PMI Nutrition International, Brentwood, MO, United States) and distilled water were provided *ad libitum* for the mice. Thirty-two male ICR mice (6 weeks old) were purchased from BioLASCO (Yi-Lan, Taiwan). After a 2-week acclimation period, all the mice were randomly divided into four groups (8 mice/group) for PL-02 supplementation and/or resistance training (RT) as follows: (1) sedentary control with vehicle (SC), (2) sedentary control with PL-02 supplementation (PL-02, 2.05 × 10^9^ CFU/kg mouse/day), (3) resistance training with vehicle (RT), and (4) resistance training with PL-02 supplementation (PL-02 + RT, 2.05 × 10^9^ CFU/kg mouse/day). Each group was administered the same volume of distilled water or ISP by oral gavage. We recorded water consumption, food intake, and animal weights twice per week.

### Resistance Training and an Aerobic Exercise Capacity Test

The resistance training protocol was performed 5 days/week for 4 weeks, and the indicated intensity load was adjusted by individual animal weight using the protocol. The weight-loading started at 5% of body weight and increased by 25% of body weight weekly until the final weight was 100% body weight, as we previously described. In resistance training, the climbing routines involved 4 repetitions/set and 3 sets/day with 1 min rest between sets (28). The equipment was set under 5 cm of water to provide negative stimulation and increase climbing motivation. The muscular power was evaluated in terms of shortest time to climb, and the maximum number of climbs to exhaustion was used to evaluate anaerobic performance.

### Forelimb Grip Strength

Grip strength was measured through a pull rod (2 mm in diameter, 7.5 cm in length) on a low-force testing system (Model-RX-5, Aikoh Engineering, Nagoya, Japan). We gently held the top of the mouse tail to allow it to swing naturally, until the two front limbs of the mouse held the lever. Then, with the mouse’s body parallel to the device, we slightly pulled in the opposite direction, repeating 10 times, and we recorded the maximum value, as previously described (26).

### Endurance Exercise Performance Test

We used a motor-driven treadmill for rodents (model MK-680, Muromachi Kikai, Tokyo, Japan) was used to assess aerobic endurance performance, and a shock grid to increase test motivation through veterinary monitoring. All mice were initially fit to run on a motorized treadmill at a 10 m/min, 5% incline for 5 min per day for 1 week prior to the exhaustive exercise test. In the formal test, we set a fixed 15° inclination angle and an initial speed of 15 m/min for the mouse to run on the treadmill, and we increased the speed by 3 m/min every 2 min. When the mouse fell into the impact zone multiple times and stayed there for more than 5 s, we defined it as exhausted (29).

### Determination of Fatigue-Associated Serum Biomarkers

Referring to our previous study, we performed a fatigue biochemical assessment in this trial to explore the benefits of PL-02 supplementation in combination with resistance training in reducing post-exercise fatigue. We collected blood from the submandibular hemorrhage of the mice immediately after swimming in water in a non-weight-bearing fashion for 15 min at a water temperature of 30°C. The blood samples were centrifuged at 1,000 × g and 4°C for 15 min after complete clotting for serum separation and analyzed using an automatic analyzer (Hitachi 7,060, Hitachi, Tokyo, Japan) to measure glucose, lactate, blood urine nitrogen (BUN), creatine kinase (CK), and ammonia levels.

### Clinical Biochemical Profiles

At the end of the experiment, we euthanized all the mice with 95% CO_2_ and collected blood samples *via* the heart. The serum was collected after centrifugation and assessed by using an automatic analyzer (Hitachi 717, Hitachi, Tokyo, Japan) for the levels of alanine aminotransferase (ALT), aspartate aminotransferase (AST), albumin (ALB), total cholesterol (TC), triacylglycerol (TG), BUN, creatinine (CREA), uric acid (UA), total protein (TP), CK, lactate dehydrogenase (LDH), and glucose.

### Body Composition and Glycogen Content Analysis

After the mice were euthanized, the liver, kidney, heart, lung, muscle (gastrocnemius), quadriceps, epididymal fat pad (EFP), and brown adipocyte tissue (BAT) were accurately excised and weighed. Of these, 100 μg of liver and muscle tissue was homogenized in 500 μL of cold perchloric acid, then centrifuged at 15,000 × g for 15 min at 4°C, the supernatant was collected, and the glycogen concentration was determined. We determined the glycogen content (mg/g) in the liver and muscle using a commercial assay kit (Sigma-Aldrich, St. Louis, MO, United States) according to the manufacturer’s instructions.

### Pathological Histology of Tissues Staining

The liver, kidney, muscle, quadriceps, heart, lung, EFP, and BAT were fixed in 10% formalin, embedded in paraffin, and cut into 4 μm-thick sections for morphological and pathological evaluation. The tissue sections were stained with hematoxylin and eosin (H&E) and examined by a clinical pathologist for light microscopy using a CCD camera (BX-51, Olympus, Tokyo, Japan).

### Statistical Analysis

The statistical analyses were performed using SAS v9.0 (SAS, Cary, NC, United States). Two-way ANOVA was used to assess the effect of the RT and PL-02 supplementation on all the experimental data. The data are expressed as mean ± SD, and *p* < 0.05 was considered statistically significant.

## Results

### General Characteristics of PL-02 Supplementation Combined With Resistance Training

As shown in Table 1, we found no significant differences in diet, water intake, or body weight in the 4 weeks of PL-02 supplementation combined with RT. In terms of body composition, both in absolute and relative weight, liver, kidney, heart, lung, EFP, and BAT showed no significant differences between the groups. However, the absolute and relative muscle weights in the PL-02 + RT group was significantly higher than in the SC group [1.16-fold (*p* = 0.0017) and 1.13-fold (*p* = 0.0030), respectively]. The main effect of PL-02 supplementation was a significant change in the absolute (*p* = 0.0046) and relative (*p* = 0.0117) muscle weights. We obtained a similar result for the quadriceps: the PL-02 + RT group showed a significant increase compared to the SC group [1.23-fold (*p* = 0.0337) and 1.10-fold (*p* = 0.0137), respectively]. The main effect of PL-02 supplementation was a significant change in the absolute (*p* = 0.0470) and relative (*p* = 0.0284) quadriceps weights.

**TABLE 1:** General characteristics of the experimental groups.

Characteristics	SC	PL-02	RT	PL-02 + RT	Main factor *p*-value		
					RT	PL-02	PL-02 + RT
Initial BW (g)	32.4 ± 0.9	32.3 ± 0.9	32.4 ± 1.1	32.4 ± 0.8	0.8940	0.8940	0.8342
Final BW (g)	37.7 ± 1.2	37.3 ± 1.3	37.1 ± 1.4	37.4 ± 1.4	0.5071	0.8268	0.4636
Water intake (ml/mouse/day)	9.45 ± 1.85	9.30 ± 1.16	9.34 ± 1.35	9.39 ± 1.08	0.9647	0.8405	0.6732
Diet intake (g/mouse/day)	7.50 ± 1.37	7.58 ± 1.24	7.60 ± 1.79	7.62 ± 1.70	0.7759	0.8472	0.9165
Liver (g)	2.06 ± 0.09	2.06 ± 0.10	2.04 ± 0.05	2.06 ± 0.08	0.8315	0.7658	0.7985
Kidney (g)	0.63 ± 0.05	0.62 ± 0.03	0.63 ± 0.07	0.62 ± 0.05	0.8950	0.6448	0.8950
quadriceps (g)	0.52 ± 0.05^a^	0.54 ± 0.04^ab^	0.53 ± 0.04^a^	0.57 ± 0.03^b^	0.2892	0.0470	0.3272
Muscle (g)	0.39 ± 0.02^a^	0.41 ± 0.04^ab^	0.40 ± 0.02^a^	0.43 ± 0.02^b^	0.0754	0.0046	0.5432
Heart (g)	0.19 ± 0.02	0.19 ± 0.01	0.19 ± 0.01	0.18 ± 0.01	0.5623	0.5623	0.1386
Lung (g)	0.23 ± 0.03	0.22 ± 0.02	0.22 ± 0.04	0.22 ± 0.03	0.5287	0.6058	0.6878
BAT (g)	0.34 ± 0.02	0.10 ± 0.02	0.10 ± 0.01	0.10 ± 0.03	0.9297	0.9297	0.3358
EFP (g)	0.34 ± 0.04	0.29 ± 0.10	0.32 ± 0.02	0.29 ± 0.08	0.7222	0.1769	0.6119
Relative liver weight (%)	5.47 ± 0.31	5.53 ± 0.37	5.52 ± 0.28	5.52 ± 0.29	0.8237	0.7978	0.7978
Relative kidney weight (%)	1.67 ± 0.16	1.67 ± 0.09	1.71 ± 0.19	1.67 ± 0.15	0.7564	0.6878	0.6878
Relative quadriceps weight (%)	1.39 ± 0.15^a^	1.45 ± 0.09^ab^	1.41 ± 0.07^a^	1.53 ± 0.10^b^	0.1704	0.0284	0.4474
Relative muscle weight (%)	1.03 ± 0.06^a^	1.10 ± 0.12^ab^	1.07 ± 0.07^a^	1.16 ± 0.06^b^	0.0675	0.0117	0.7927
Relative heart weight (%)	0.49 ± 0.04	0.51 ± 0.04	0.52 ± 0.04	0.48 ± 0.04	0.7509	0.6183	0.0589
Relative lung weight (%)	0.62 ± 0.06	0.60 ± 0.05	0.60 ± 0.11	0.59 ± 0.10	0.6882	0.6274	0.8491
Relative BAT weight (%)	0.25 ± 0.04	0.27 ± 0.05	0.27 ± 0.03	0.26 ± 0.07	0.9739	0.9739	0.2859
Relative EFP weight (%)	0.89 ± 0.12	0.77 ± 0.26	0.85 ± 0.05	0.78 ± 0.20	0.8020	0.1425	0.6521

*Data are expressed as mean ± SD and the same row with different letters (a,b) differ significantly at p < 0.05 by two-way ANOVA. EFP, epididymal fat pad; BAT, brown adipose tissue.*

### The Effect of 4 Weeks PL-02 Supplementation Combined With Resistance Training on Grip Strength

**FIGURE 1:**
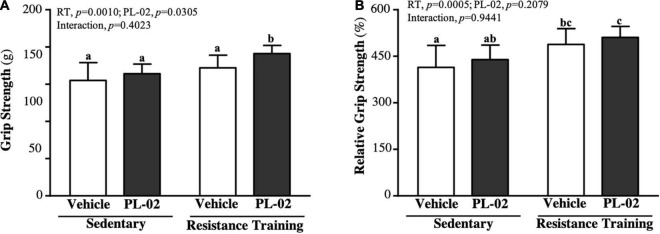
Effect of PL-02 supplementation combined with RT on **(A)** absolute forelimb grip strength and **(B)** forelimb grip strength (%) relative to body weight. Data are expressed as mean ± *SD* and different superscript letters (a,b,c) indicate significant difference at *p* < 0.05.

As shown in Figure 1A, the SC, PL-02, RT, and PL-02 + RT groups were 155 ± 24, 164 ± 13, 172 ± 17 and 191 ± 1 g, respectively. The PL-02 + RT group was significantly heavier than the SC, PL-02 and RT groups [1.23-fold (*p* = 0.0002), 1.17-fold (*p* = 0.0033), and 1.11-fold (*p* = 0.0353), respectively]. The main effect of RT (*p* = 0.0010) and PL-02 (*p* = 0.0305) was a significant increase in grip strength, but we found no significant interaction effect. We calculated the relative grip strength (%) normalized to body weight, and the RT and PL-02 + RT groups scored significantly higher than the SC group [1.18-fold (*p* = 0.0086) and 1.23-fold (*p* = 0.0010), respectively], with only significant RT main effects (*p* = 0.0005) (Figure 1B).

### The Effect of 4 Weeks PL-02 Supplementation Combined With Resistance Training on Anaerobic Exercise Performance

**FIGURE 2:**
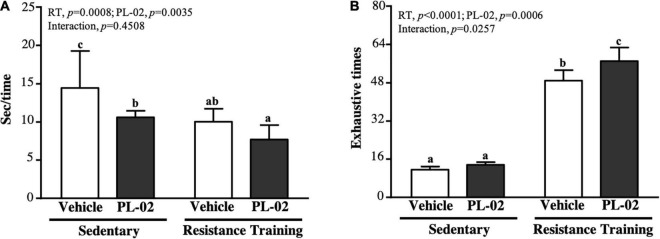
Effect of PL-02 supplementation combined with RT on **(A)** time for each climbing and **(B)** exhaustion times for climbing. Data are expressed as mean ± *SD* and different superscript letters (a,b,c) indicate significant difference at *p* < 0.05.

Speed and muscular endurance were used as measures of anaerobic exercise performance, as shown in Figure 2A. The speed performance in the SC, PL-02, RTm and PL-02 + RT groups were 14.46 ± 4.80, 10.61 ± 0.86, 10.05 ± 1.69 and 7.69 ± 1.91 s, respectively. The PL-02, RT and PL-02 + RT groups scored significantly lower than the SC group by 26.62% (*p* = 0.0092), 30.47% (*p* = 0.0034), and 46.80% (*p* < 0.0001), respectively. The main effect of RT (*p* = 0.0008) and PL-02 (*p* = 0.0035) was a significant increase in climbing speed, but we found no significant interaction effect. The repetition maximum (RM) of the SC, ISP, RT, and ISP + RT groups was 11.6 ± 1.3, 13.6 ± 1.1, 48.9 ± 4.4, and 57.0 ± 5.7 times, respectively. The RMs of the RT and ISP + RT groups were significantly higher than those of the SC group [4.20-fold (*P* = 0.0027) and 4.90-fold (*P* < 0.0001), respectively]. The main effect of RT (*p* < 0.0001) and PL-02 (*p* = 0.0006) was a significantly increased muscular endurance performance, and they had a significant interaction effect (*p* = 0.0257) (Figure 2B).

### The Effect of 4 Weeks PL-02 Supplementation Combined With Resistance Training on Endurance Exercise Performance

**FIGURE 3:**
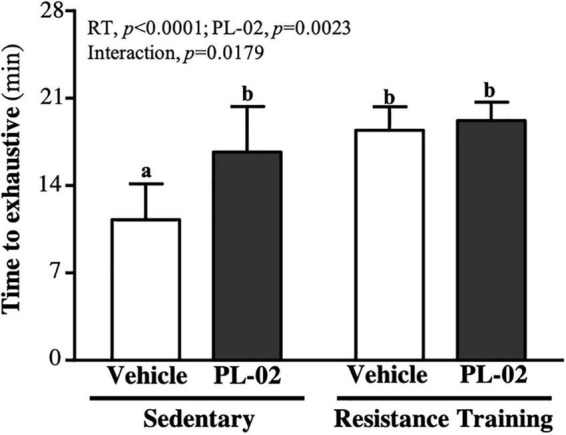
Effect of PL-02 supplementation combined with RT on endurance exercise performance. Data are expressed as mean SD and different superscript letters (a,b) indicate significant difference at *p* < 0.05.

As shown in Figure 3, the treadmill running times to exhaustion in the SC, PL-02, RT, and PL-02 + RT groups were 11.27 ± 2.87, 16.69 ± 3.66, 18.44 ± 1.87, and 19.21 ± 1.47 (min), respectively. Compared to the SC group, the PL-02, RT and PL-02 + RT groups scored significantly higher [1.48-fold (*p* = 0.0008), 1.64-fold (*p* = 0.0002), and 1.70-fold (*p* < 0.0001), respectively]. The main effect of RT (*p* < 0.0001) and PL-02 (*p* = 0.0023) was a significant increase in muscular endurance performance, and we found a significant interaction effect (*p* = 0.0179).

### The Effect of 4 Weeks PL-02 Supplementation Combined With Resistance Training on Fatigue-Related Biochemical Parameters After the 15-min Swim Test

**FIGURE 4:**
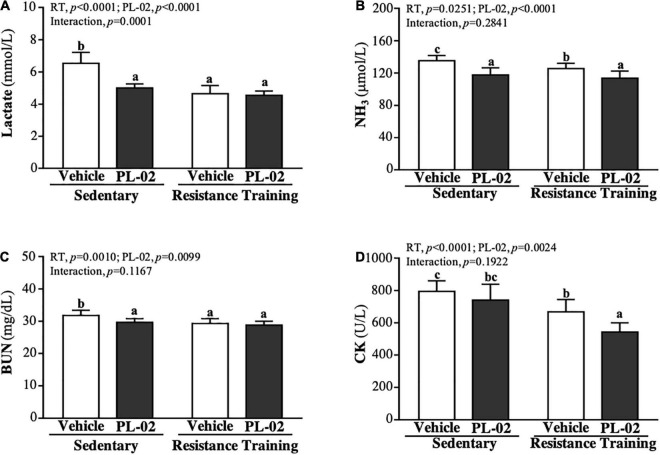
Effect of PL-02 supplementation combined with RT on **(A)** lactate, **(B)** NH_3_, **(C)** BUN and **(D)** CK after 15 min swimming. Data are expressed as mean ± *SD* and different superscript letters (a,b,c) indicate significant difference at *p* < 0.05.

After 4 weeks’ PL-02 supplementation combined with RT, we conducted 15 min of acute exercise with each mouse to explore the effects on the biochemical markers of fatigue. The lactate levels (Figure 4A) of the SC, PL-02, RT, and PL-02 + RT groups were 6.53 ± 0.68, 5.00 ± 0.25, 4.65 ± 0.50, and 4.55 ± 0.26 (mmol/L), respectively. Compared to the SC group, the lactate levels of the PL-02, RT and PL-02 + RT groups showed significant decreases of 23.39% (*p* < 0.0001), 28.86% (*p* < 0.0001), and 30.37% (*p* < 0.0001), respectively. As shown in Figure 4B, the NH_3_ levels in the SC, PL-02, RT, and PL-02 + RT groups were 169 ± 8, 147 ± 11, 157 ± 8, and 142 ± 11 (μmol/L), respectively, with the levels in the RT and PL-02 + RT groups showing significant decreases of 15.85% (*p* = 0.0025) and 31.63% (*p* < 0.0001) compared to the SC group, respectively. The BUN levels in the SC, PL-02, RT, and PL-02 + RT groups were 31.8 ± 1.6, 29.7 ± 1.1, 29.3 ± 1.5, and 28.8 ± 1.2 (mg/dL), respectively. Compared to the SC group, the BUN levels in the PL-02, RT and PL-02 + RT groups showed significantly decreases of 6.60% (*p* = 0.0044), 7.97% (*p* = 0.0008), and 9.70% (*p* < 0.0001), respectively (Figure 4C). As for CK activity, the SC, PL-02, RT and PL-02 + RT groups scored 794 ± 66, 740 ± 98, 668 ± 76, and 543 ± 57 (U/L), respectively. The RT and PL-02 + RT groups showed significant decreases in the CK activity of 15.85% (*p* = 0.0025) and 31.63% (*p* < 0.0001), respectively (Figure 4D). The main effect of RT and PL-02 was a significant decrease in lactate, NH3, BUN, and CK levels (*p* < 0.05), but only the decrease in the lactate level had a significant interaction effect (*p* < 0.0001).

### The Effect of 4 Weeks PL-02 Supplementation Combined With Resistance Training on Tissues Glycogen Content

**FIGURE 5:**
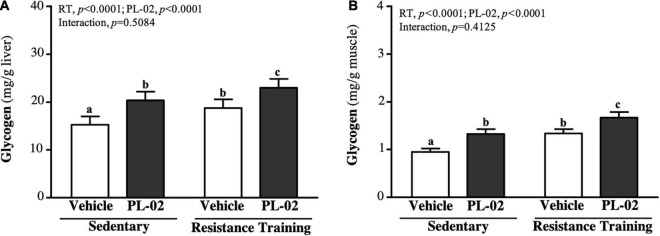
Effect of PL-02 supplementation combined with RT on **(A)** liver glycogen and **(B)** muscle glycogen. Data are expressed as mean SD and different superscript letters (a,b,c) indicate significant difference at *p* < 0.05.

Glycogen is mainly stored in the liver and skeletal muscle. The liver glycogen levels in the SC, PL-02, RT, and PL-02 + RT groups were 15.30 ± 1.71, 20.39 ± 1.82, 18.78 ± 1.82, and 23.02 ± 1.83 (mg/g), respectively. The levels in the PL-02, RT, and PL-02 + RT groups showed significant increases compared to the SC group [1.33-fold (*p* < 0.0001), 1.23-fold (*p* = 0.0006), and 1.50-fold (*p* < 0.0001), respectively; Figure 5A]. As shown in Figure 5B, the muscle glycogen levels in the SC, PL-02, RT, and PL-02 + RT groups were 0.95 ± 0.07, 1.33 ± 0.10, 1.34 ± 0.09, and 1.67 ± 0.12 (mg/g), respectively. The muscle glycogen levels in the PL-02, RT, and PL-02 + RT groups were significantly increased compared to that of the SC group [1.41-fold (*p* < 0.0001), 1.41-fold (*p* < 0.0001), and 1.76-fold (*p* < 0.0001), respectively]. The main effect of RT and PL-02 was a significant increase in liver and muscle glycogen (*p* < 0.0001), but neither of them showed a significant interaction effect.

### The Effect of 4 Weeks PL-02 Supplementation Combined With Resistance Training on Biochemical Variables at the End of the Experiment

At the end of the experiment, a clinical biochemical analysis was performed on the serum, as shown in Table 2. We found no significant differences in ALB, TC, TG, CREA, UA, TP, CK, LDH, or glucose levels. However, the AST and ALT activity levels in the RT group were significantly lower than in the sedentary groups, being the main effect of exercise training (*p* < 0.0001).

**TABLE 2:** The effect of PL-02 supplementation combined with RT on biochemical assessments of serum at the end of the experiment.

Characteristics	SC	PL-02	RT	PL-02 + RT	Main factor *p*-value		
					RT	PL-02	RT + PL-02
AST (U/L)	126 ± 7^b^	127 ± 13^b^	92 ± 6^a^	92 ± 6^a^	< 0.0010	0.9835	0.9505
ALT (U/L)	44 ± 12^b^	43 ± 8^b^	38 ± 8^ab^	34 ± 6^a^	0.0230	0.4374	0.5662
TC (mg/dL)	135 ± 13	133 ± 14	136 ± 10	137 ± 8	0.4886	0.8894	0.7225
TG (mg/dL)	102 ± 9	106 ± 6	101 ± 7	106 ± 5	0.8440	0.0931	1.0000
CK (U/L)	390 ± 67	379 ± 64	380 ± 52	362 ± 43	0.5173	0.4903	0.8715
BUN (mg/dL)	25.3 ± 6.2	25.3 ± 2.0	23.6 ± 1.7	23.3 ± 2.2	0.1476	0.9443	0.9206
CREA (mg/dL)	0.38 ± 0.02	0.39 ± 0.02	0.39 ± 0.02	0.39 ± 0.01	0.4287	0.9295	0.9295
UA (mg/dL)	1.6 ± 0.3	1.6 ± 0.6	1.6 ± 0.4	1.5 ± 0.3	0.9670	0.9014	0.9670
ALB (g/dL)	3.0 ± 0.1	3.0 ± 0.1	3.0 ± 0.1	3.0 ± 0.1	0.7890	0.6993	0.9053
TP (g/dL)	5.7 ± 0.3	5.6 ± 0.3	5.6 ± 0.2	5.6 ± 0.2	0.7331	0.5403	0.3103
Glucose (mg/dL)	191 ± 12	194 ± 16	208 ± 18	205 ± 11	0.0145	0.9904	0.5573

*Data are expressed as mean ± SD and the same row with different letters (a,b) differ significantly at p < 0.05 by two-way ANOVA.*

*AST, aspartate aminotransferase; ALT, alanine aminotransferase; TC, total cholesterol; TG, triglycerides; CK, creatine kinase; ALB, albumin; BUN, blood urea nitrogen; CREA, creatinine; UA, urea acid; TP, total protein.*

### The Effect of 4 Weeks PL-02 Supplementation Combined With Resistance Training on Histological Observation

**FIGURE 6:**
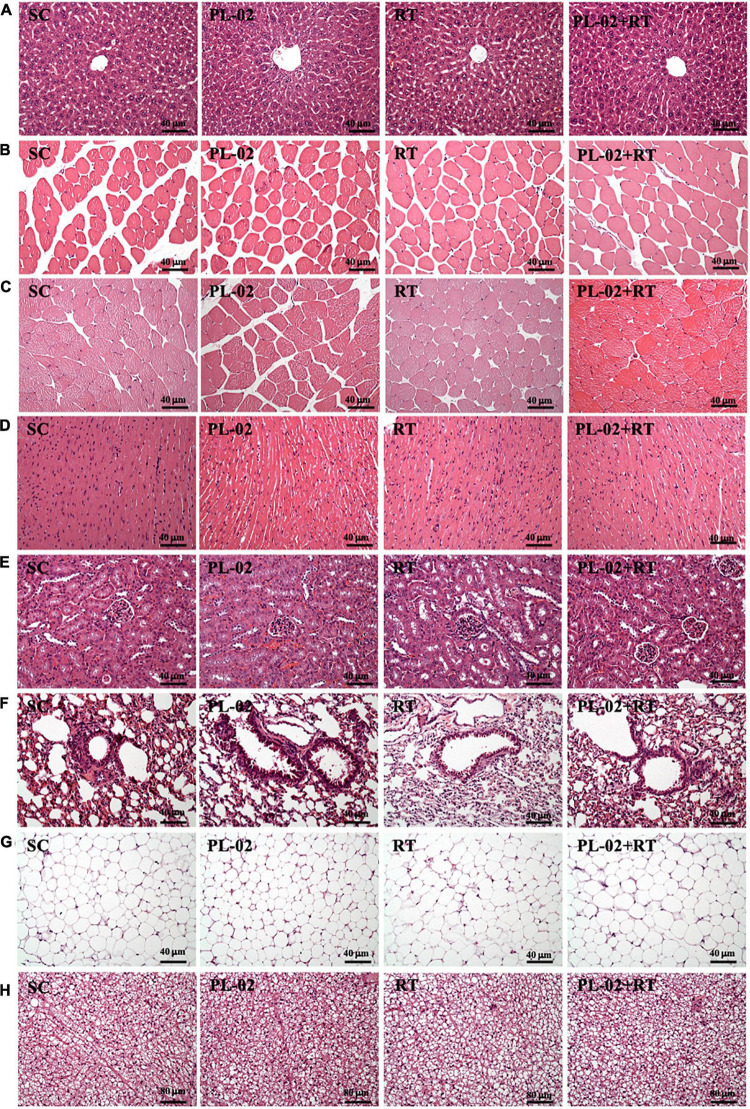
Effect of PL-02 supplementation combined with RT on **(A)** liver, **(B)** quadriceps, **(C)** muscles, **(D)** heart, **(E)** kidney, **(F)** lung, **(G)** EFP, and **(H)** BAT tissue in mice. (H&E stain, magnification: 200×; bar, 40 μm; BAT magnification: 100×; bar, 80 μm). EFP, epididymal fat pad; BAT, brown adipose tissue.

Histological examinations of the liver, quadriceps, muscle, heart, kidney, lung, EFP, and BAT were performed at the end of the study, and no abnormalities were observed in any group (Figure 6). Intrahepatic sinusoidal and hepatic cord arrangement did not change. In addition, no Zenker degeneration or hyperplasia was observed in cardiomyocytes, and we found no differences in tubular or glomerular structure between treatment groups.

## Discussion

In recent years, a growing body of researchers has explored the role of probiotics in improving athletic performance. However, literature on the use of probiotics as a sports nutrition supplement in combination with exercise training is scarce. In the current study, we found that *L. plantarum* PL-02 supplementation was effective in improving muscle mass, muscle strength, and exercise endurance performance, and in increasing glycogen content, consistent with previous findings (27). In addition, the effect when combined with RT intervention was more significant.

RT is the primary form of exercise for building muscle health regardless of diet and nutrition. Regular resistance exercise can enhance the phosphorylation of p70S6 kinase and MPS, promoting significant increases in muscle strength and hypertrophy (30). This works by causing microscopic damage or tearing to muscle cells, which in turn are quickly repaired by the body to help the muscle regenerate and become stronger (31). Furthermore, higher proportions of SCFA bacteria involved in carbohydrate and amino acid metabolism in the gut of athletes lead to higher concentrations of acetate, butyrate, and propionate in feces (32). Among them, acetate enhances glucose uptake and fatty acid metabolism by activating activated protein kinase (AMPK) and increasing the expression of glucose transporter type 4 (GLUT4) and myoglobin. Myocyte enhancer factor 2A (MEF2A), a metabolic pathway thought to be important for increasing muscle mass, is involved in the expression of myoglobin and GLUT4 genes (33). Butyrate may increase ATP and the metabolic efficiency of muscle fibers by activating pathways such as UCP2-AMPK-ACC and PGC1-α (34). In addition, it prevents apoptosis and prevents muscle protein catabolism by inhibiting histone deacetylases (35). In addition to increasing the production of SCFA in the gut, probiotic supplementation is effective in increasing the alpha diversity in the gut and may affect muscle mass, function, and energy metabolism by altering the microbiota. In our previous study, mice administered TWK10 or PL-02 for 6 or 4 weeks, respectively, showed increased muscle mass without exercise training (22, 27). In this study, after 4 weeks of PL-02 supplementation with resistance exercise training, muscle mass significantly increased (Table 1). Increased muscle mass positively correlates with improved muscle strength, which increases with muscle mass (36). We found similar trend results in this study. Supplementation with PL-02 in combination with RT significantly improved muscle strength and relative muscle strength (Figures 1A,B). Increases in muscle strength generally increase explosive power, and explosive strength training can help improve muscular endurance and endurance performance (37). In this study, we measured the shortest crawl time (explosive power) and the maximum number of crawls (muscular endurance). The results showed that PL-02 supplementation in combination with RT had a significant effect not only on improved muscle mass and strength, but also on explosive power and muscular endurance performance (Figures 2A,B).

Muscle mass and quantity, as well as mitochondrial mass, affect glycogen content (38). Glycogen is a glucose polymer that produces adenosine triphosphate (ATP) through glycogen catabolism and provides the main source of fuel for muscles during exercise (39). If glycogen stores are reduced, bioenergy metabolism is impaired, resulting in insufficient energy supply and decreased muscle strength and function (40). Previous study had shown that, when the gut microbiota is dysregulated, skeletal muscle glucose availability may be reduced, resulting in reduced glycogen storage (13). Germ-free mice showed significantly lower glycogen stores compared to mice with a normal gut microbiota (41). The strains in the gut may produce SCFA, in which butyrate can maintain blood glucose homeostasis and promote glycogen metabolism through the GPR43–AKT–GSK3 signaling pathway (42), whereas propionate can promote gluconeogenesis in hepatocytes (43). Supplementation with probiotics can increase the production of SCFAs in the gut. *Lactobacillus acidophilus* modulates glycogen-synthesis-related genes (GSK-3β and Akt) and glycogen content in tissues (44). In our previous study, we also found that *Bifidobacterium longum* OLP-01 supplementation for four consecutive weeks significantly increased glycogen levels in the muscle and liver of mice and improved exercise endurance performance (23). In this study, either RT or PL-02 supplementation significantly increased liver or muscle glycogen content, but the effect of RT combined with PL-02 intervention was more significant (Figures 5A,B). Optimizing and increasing glycogen storage can effectively improve exercise endurance performance, thereby delaying post-exercise fatigue and accelerating recovery (45). In addition, the modulation of SCFA production by the gut microbiota affects energy metabolism during exercise, thereby contributing to exercise-induced adaptation and serving as an energy source for liver and muscle cells, enhancing endurance performance using the long-term maintenance of blood glucose (46). Our previous findings showed that supplementation with PL-02 can significantly increase the *A. muciniphila* in the gut (27), which is thought to have a higher proportion in the gut microbiota of athletes. Maintaining gut barrier function and glucose homeostasis can help sustain or enhance endurance performance (47). In the current study, RT or PL-02 supplementation had a significant effect on improving exercise endurance, and we observed an interaction effect (Figure 3).

During prolonged or strenuous exercise, the body is unable to provide or maintain the required energy load, resulting in decreased exercise performance and an increased production of fatigue byproducts (47). Among them, exercise-related indicators such as lactate, NH3, BUN, and CK are widely used to evaluate the physiological state of exercise. Lactate, a product of carbohydrate glycolysis under anaerobic conditions, is positively correlated with exercise duration and intensity. During vigorous exercise, glucose is broken down into pyruvate. Under anaerobic conditions, a part of pyruvate is reduced to lactic acid by lactate dehydrogenase (LDH) and hydrogen ions are released, resulting in a decrease in the pH of blood and muscle tissue and the inhibition of glycolysis, which in turn interferes with normal cell function (48, 49). In order to maintain the ATP/ADP ratio, two molecules of ADP can be converted into one molecule of ATP and one molecule of AMP when the ATP supply is insufficient to cope with exercise consumption. Among them, AMP is degraded into IMP and ammonia by AMP deaminase. Ammonia is converted to BUN through the urea cycle, thereby increasing in blood (50). Long-term exercise training can significantly increase the intestinal level of *Veillonella*. *Veillonella* species metabolize lactate to SCFA acetate and propionate *via* the methylmalonyl–CoA pathway (51). In addition, the intestinal colonization of *Veillonella* may enhance the Cori cycle by providing an alternative lactate disposal method that converts systemic lactate into SCFAs that re-enter the circulation (52). Probiotic supplementation increases SCFAs in the gut and converts lactic acid to propionate, which provides energy for muscles during exercise (53). The mechanism of these synergistic effects may involve the increased availability of glycogen or glucose fuel (54). Therefore, these results were validated in our research. In our study, RT and PL-02 supplementation had a significant effect on reduced post-exercise lactate, NH3, BUN, and CK (Figures 4A–D).

Resistance training has been shown to improve gut microbiota diversity and composition and to help increase the richness of SCFA-producing gut microbiota and reduce the relative abundance of pro-inflammatory-inducing species, including *Pseudomonas, Serratia, Comamonas*, etc. (55, 56). A past study showed that elite rugby players had higher gut microbial diversity compared to sedentary people, and with a significantly higher percentage of *Akkermansia* (32). *A. muciniphila*, a gut microbe known for mucin degradation and SCFA production, is thought to be positively correlated with exercise performance in its abundance (57), in addition to maintaining gut barrier function and glucose homeostasis (46). In our previous study also confirmed that supplementation of PL-02 alone without any exercise training also significantly increased the proportion of *Akkermansia* in the mouse gut and improved muscle mass and exercise performance (27). In the current study, designed to investigate the benefits of PL-02 in combination with resistance training to improve athletic performance and muscle mass, we confirmed this result. However, whether it has a synergistic effect on gut microbial composition and changes remains to be further confirmed in the future.

We found that supplementation with PL-02 combined with RT for four consecutive weeks effectively improved muscle strength, muscle mass, endurance, and glycogen storage in mice, and significantly reduced the biochemical values of fatigue after exercise. In addition, according to the serum biochemical analysis and histopathological results, no abnormality or injury was observed even after repeated exercise challenges. However, no synergistic effect was observed between RT and PL-02 supplementation for muscle strength, muscle mass, biochemical markers of fatigue, and glycogen stores. We think that the intervention period needs to be further expanded or the training intensity or dose of probiotics needs to be increased. Therefore, we must further explore the optimal cycle and strategy of probiotics combined with exercise training intervention. In addition, in animal experiments, the administration of probiotics effectively increased muscle mass, improved exercise performance, and reduced fatigue after exercise without exercise intervention; its applicability and efficacy were also successfully demonstrated in humans (18, 19). Therefore, we infer that similar result can be achieved in human trials in the future. However, further research is needed to clarify the mechanism of human-related effects.

## Conclusion

In conclusion, we found that *L. plantarum* PL-02 supplementation combined with resistance exercise training over 4 weeks significantly improved forelimb grip, endurance exercise performance, and glycogen storage, and increased muscle mass. In addition, PL-02 + RT significantly reduced the levels of fatigue indicators such as lactate, BUN, ammonia, and CK. Furthermore, PL-02 + RT did not cause harm to physiological performance or histopathology. Our research confirmed that PL-02 or RT supplementation alone are effective in reducing exercise fatigue and in improving muscle mass and exercise performance, but the combination of the two can achieve increased benefits.

## Data Availability Statement

The raw data supporting the conclusions of this article will be made available by the authors, without undue reservation.

## Ethics Statement

The animal study was reviewed and approved by the Institutional Animal Care and Use Committee (IACUC) of the National Taiwan Sport University approved the animal experimentation and procedure (IACUC no. 11008).

## Author Contributions

W-LY, Y-JH, and C-CH designed the study. Y-JH, C-SH, and M-CL carried out the experiments. W-LY, C-SH, C-CH, and M-CL analyzed the data. H-HH, W-YL, Y-WK, S-YT, and C-CH provided reagents and other lab supplies. W-LY, Y-JH, C-CH, and M-CL prepared the figures and wrote the manuscript. W-LY, C-CH, and M-CL revised the manuscript. All authors contributed to the article and approved the submitted version.

## Conflict of Interest

H-HH, Y-WK, and S-YT were employed by Bioflag Biotech Co., Ltd. The remaining authors declare that the research was conducted in the absence of any commercial or financial relationships that could be construed as a potential conflict of interest.

## Publisher’s Note

All claims expressed in this article are solely those of the authors and do not necessarily represent those of their affiliated organizations, or those of the publisher, the editors and the reviewers. Any product that may be evaluated in this article, or claim that may be made by its manufacturer, is not guaranteed or endorsed by the publisher.
